# Effective ambiguity checking in biosequence analysis

**DOI:** 10.1186/1471-2105-6-153

**Published:** 2005-06-20

**Authors:** Janina Reeder, Peter Steffen, Robert Giegerich

**Affiliations:** 1 InternationaI NRW Graduate School of Bioinformatics and Genome Research, Center of Biotechnology (CeBiTec), Bielefeld University, Postfach 10 01 31, 33501 Bielefeld, Germany; 2 Practical Computer Science, Faculty of Technology, Bielefeld University, Postfach 10 01 31, 33501 Bielefeld, Germany

## Abstract

**Background:**

Ambiguity is a problem in biosequence analysis that arises in various analysis tasks solved via dynamic programming, and in particular, in the modeling of families of RNA secondary structures with stochastic context free grammars. Several types of analysis are invalidated by the presence of ambiguity. As this problem inherits undecidability (as we show here) from the namely problem for context free languages, there is no complete algorithmic solution to the problem of ambiguity checking.

**Results:**

We explain frequently observed sources of ambiguity, and show how to avoid them. We suggest four testing procedures that may help to detect ambiguity when present, including a *just-in-time *test that permits to work safely with a potentially ambiguous grammar. We introduce, for the special case of stochastic context free grammars and RNA structure modeling, an automated partial procedure for proving non-ambiguity. It is used to demonstrate non-ambiguity for several relevant grammars.

**Conclusion:**

Our mechanical proof procedure and our testing methods provide a powerful arsenal of methods to ensure non-ambiguity.

## Background

### The ambiguity problem in biosequence analysis

Biosequence analysis problems are typically optimization problems – we seek the best alignment of two protein sequences under a similarity score, or the most stable secondary structure of an RNA molecule under a thermodynamic model. In such a problem, there is a "good" and a "bad" type of ambiguity. The good one is that there are many solutions to choose from. The bad one is that our algorithm may find the same solution several times, or even worse, it may study seemingly different solutions, which in fact represent the same object of interest. The cause of all these phenomenona has been called ambiguity, because it is closely related to the ambiguity problem of formal languages. It is not quite the same problem, however. In striving for avoidance of ambiguity, we want to get rid of the bad type and retain the good.

Ambiguity is not a problem with a dynamic programming (DP) algorithm that returns a single, optimal score, together with a solution that achieves this score, and does not make assertions about other solutions in the search space. Then, it does not matter whether this solution is analyzed several times, or that there are other solutions achieving the optimal score. In other cases, ambiguity can cause a DP algorithm to return an "optimal" answer which is plainly wrong. In the presence of ambiguity, the Viterbi algorithm cannot report the most likely structure [1], a folding program cannot produce a complete and non-redundant set of suboptimal structures [2], and statistics like counts, sum over all scores (by an Inside-type algorithm), or expected number of feasible or canonical structures [3] cannot be computed.

### Previous work

The phenomenon of ambiguity has been formalized and studied in [3] in a quite general framework of dynamic programming over sequence data. There, it is shown that for a proof of non-ambiguity, a canonical model of the studied domain is required. The canonical model plays an essential role. It is the mathematical formalization of the real-world domain we want to study, and "canonical" means one-to-one correspondence. Any formal proof can only deal with the formalization of the real-world domain, and when the one-to-one correspondence does not hold, all proofs of (non-) ambiguity would be meaningless for the real world. In general, it may be quite difficult to find a canonical model for some real-world domains. Our case, however, is easy. When RNA secondary structure is our domain of study, base pair sets or the familiar dot-bracket strings can serve as a canonical model, as they uniquely represent secondary structures. To ensure non-ambiguity, there must exist an injective (i.e. one-to-one) mapping from derivation trees (according to the grammar underlying the DP algorithm) to the canonical model. While such a mapping may be easy to specify, the proof of its injectivity remains a problem.

Recently, Dowell and Eddy have re-addressed this problem [1] in the framework of stochastic context free grammars (SCFGs). In a probabilistic framework, ambiguity matters when a best, i.e. most likely solution is computed. This solution is wrong if several "different" solutions represent the same real-world object. Dowell and Eddy experimented with two ambiguous SCFGs, and showed that the quality of results may range from just slightly wrong to totally useless. After having shown that one cannot get by with ignoring ambiguity, they provide four non-ambiguous SCFGs for RNA structure analysis; however, a proof of their non-ambiguity is not given. Instead, they suggest a testing approach to check for the presence of ambiguity, which, of course, cannot prove its absence.

In this contribution, we first review the ambiguity problem in the framework of SCFG modeling, explain some of its sources, prove its algorithmic undecidability, and suggest three ways to deal with it: ambiguity avoidance, testing for ambiguity, and, best of all when successful, a mechanical proof of absence.

### Formalization of ambiguity

We formalize the problem at hand in two steps, going from context free grammars (CFGs) to stochastic context free grammars, and then differentiating between syntactic and semantic ambiguity.

#### Formal grammars

A formal language is a subset of the set of all strings over a finite alphabet. Formal languages are typically described by formal grammars. In general, a formal grammar consists of an alphabet, a set of nonterminal symbols, and a set of production rules. There exist various grammar types, differing in the laws for construction of these production rules. The expressive power of a grammar type depends on these laws. In 1956, Noam Chomsky introduced a hierarchy of formal grammars that ranks grammar types by their expressive power, the Chomsky hierarchy [4]. It consists of four levels: regular grammars, context-free grammars, context-sensitive grammars, and unrestricted grammars. Here, we only address context-free grammars. These are suitable to describe the pseudoknot-free secondary structure of RNA. When considering pseudoknots, context-sensitive grammars are needed.

#### Context free grammars

A context free language is described by a context free grammar *G*, given by a set of terminal symbols (the alphabet), a set of nonterminal symbols, including a designated axiom symbol, and a set of production rules of the form *X *→ *α*, where *X *is a nonterminal symbol, and a is a string of terminal and nonterminal symbols, *α *may be the empty string, denoted *ε*. Starting with the axiom symbol, by successive replacement of nonterminal symbols by right-hand sides of corresponding productions, we can derive a set of terminal strings. They constitute the language of the grammar, denoted *L*(*G*) Without loss of generality, derivations are canonized by replacing, in each step, the leftmost nonterminal symbol in the string obtained so far. Each such derivation can uniquely be represented as a derivation tree, and if the same terminal string has two different derivation trees, the grammar is called ambiguous.

Our first example is Dowell and Eddy's grammar *G*1 [1] to describe RNA secondary structures:

*G*1: *S *→ *aSu *| *uSa *| *cSg *| *gSc *| *gSu *| *uSg*

*S *→ *aS *| *cS *| *gS *| *uS*

*S *→ *Sa *| *Sc *| *Sg *| *Su*

*S *→ *SS*

*S *→ *ε*

In the following, we shall use a shorthand notation, where *a *stands for any base A,C,G,U, while *α *and  occurring in the same rule stand for either one of the base pairs (A,U), (U,A), (C,G), (G,C), (G,U), or (U,G).

*G*1: *S *→ *aS*| *aS *| *Sa *| *SS *| *ε*

**Figure 1:**
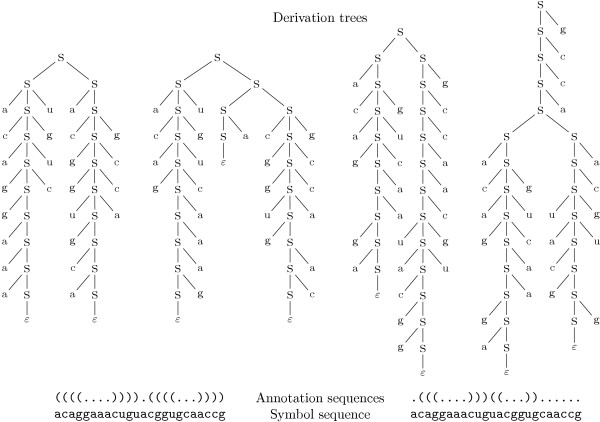
**Four derivation trees**. Four derivation trees for RNA sequence "acaggaaacuguacggugcaaccg", two (left) representing the annotation sequence ((((....)))).((((...)))) and two (right) the annotation sequence .(((....)))((...)).......

Four different derivation trees of the grammar *G*1 are shown in Figure 1. As they all emerge from the same terminal string acaggaaacuguacggugcaaccg, this grammar is ambiguous.

#### Stochastic context free grammars

Stochastic context free grammars associate a (nonzero) probability with each production, such that the probabilities for all alternative productions emerging from the same nonterminal symbol add up to 1. As a string is derived, probabilities of the involved rules multiply.

We extend the CFG *G*1 to a SCFG by the following example probabilities:

*P*_*S*→*aS*_ = 0.2

*P*_*S*→*aS *_= 0.2

*P*_*S*→*Sa *_= 0.2

*P*_*S*→*SS *_= 0.2

*P*_*S*→*ε *_= 0.2

For simplicity, we chose probabilities independent of certain bases. In SCFG design, often also non-canonical base pairings are allowed with a low probability.

For grammar *G*1, the derivations shown in Figure 1 have probabilities of 5.24·10^-14^, 2.1·10^-15^, 4.19·10^-16 ^and 4.19·10^-16 ^(from left to right).

All derivations for a string can be constructed by a CYK-type parser [5]. The parser may compute the overall probability of a given string, summing up probabilities over all its derivations, in which case it is called the Inside algorithm. Or, the parser can return the most likely derivation of the input string, in which case it is known as the Viterbi algorithm. For grammar *G*1, the corresponding CYK-based Viterbi algorithm is shown here:

Input: Sequence *x *= *x*_1 _... *x*_*n*_

Initialization: for 1 ≤ *i *≤ *n*

*S*(*i*, *i*) = *P*_*S*→*ε*_

Iteration: for 1 ≤ *i *≤ *j *≤ *n*



#### Syntactic versus semantic ambiguity

Above, we introduced the formal language-theoretic notion of ambiguity: if the same symbol sequence has two or more different derivation trees, the grammar is called ambiguous. For clarity, we will refer to it as *fl-ambiguity*. In this sense, grammar *G*1 (and every other grammar in this manuscript) is in any case fl-ambiguous. This is demonstrated by the fact that the four derivation trees of Figure 1 all belong to the same symbol sequence. We now need to refine this notion of ambiguity.

In modeling with SCFGs, derivations do not merely produce strings, but they represent objects of interest themselves. With RNA, a derivation of an RNA sequence represents a possible secondary structure of this sequence. A more compact representation of a secondary structure is the widely used dot-bracket notation, as shown at the bottom of Figure 1. In the following, we will use the term *annotation sequence *for the dot-bracket string representing one secondary structure of the underlying RNA sequence. The one-to-one correspondence between (molecular) structures and (in silico) annotation sequences qualifies the latter as a canonical model of the grammar.

By the term *syntactic ambiguity *we denote the fact that typically an RNA sequence has many secondary structures, i.e. annotation sequences, hence many derivations. Figure 1 shows two example annotation sequences of the same RNA sequence.

*Semantic ambiguity *exists when there are, for some sequence, several derivations that represent the same annotation sequence, and hence, the same secondary structure. This is our point of study. In this case, the probability of a certain annotation sequence is split up into the probabilities of its multiple derivations. In Figure 1, this is exemplified by the two derivations on the left that both represent the annotation sequence ((((....)))).((((...)))), and the two derivations on the right, that both represent the annotation sequence .(((....)))((...))....... Thus, grammar *G*1 is syntactically as well as semantically ambiguous.

Semantic ambiguity is the "bad", syntactic ambiguity the "good" type of ambiguity in SCFG modeling and dynamic programming that was mentioned above. On the pure formal language level, they cannot be distinguished – both are manifest as fl-ambiguity. The bad ambiguity hides with the good, which is why its presence is sometimes overlooked.

Semantic ambiguity is not a problem with the Inside algorithm, as a probability sum over all derivations is computed anyway. With the Viterbi algorithm, we can certainly obtain the most likely derivation, but we do not know whether it represents the most likely annotation sequence. Some other annotation sequence may be more likely, but as its probability is the sum of many different derivations, none of these derivations may come out optimal. And even if the most likely annotation sequence is returned by the Viterbi algorithm, its computed probability is too small when there are further derivations of this annotation sequence.

As Dowell and Eddy have shown, this happens in practice and the effects are severe. For correct modeling with SCFGs, we need grammars that are syntactically, but not semantically ambiguous.

#### Semantic ambiguity in dynamic programming

Our treatment here extends to all dynamic programming algorithms that fall into the class known as algebraic dynamic programming (ADP) [6]. However, some definitions must be refined, as the ADP approach uses so-called yield grammars rather than (S)CFGs. We will not introduce the ADP formalism here, but remain within the SCFG terminology. Still, we shall refer to some DP algorithms that are not based on SCFGs, where our treatment also applies.

### SCFGs for RNA secondary structure analysis

We will further exemplify the above using the grammars *G*1 to *G*6 studied by Dowell and Eddy:



Dowell and Eddy showed that grammars *G*1 and *G*2 are semantically ambiguous, while *G*3 to *G*6 passed a partial test for non-ambiguity.

## Results and discussion

In this section, we first review some sources of ambiguity and suggest three ways to deal with it: ambiguity avoidance, testing for ambiguity, and, best of all when successful, a mechanical proof of absence.

### Sources of ambiguity, and how to avoid them

We first study some standard patterns that give rise to ambiguity in our grammars. Thereafter, we make some observations with respect to the potential of testing procedures.

#### Three simple cases

Ambiguity does not sneak into our grammars by chance and non-awareness. There are two competing goals in grammar design, and both may foster ambiguity.

Small grammars have the advantage that they require fewer parameters and can be trained more quickly. Larger grammars allow a more sophisticated distinction of cases, hence providing a more fine-tuned model. However, if the underlying "distinct" cases lead to the same annotation sequence, then the grammar is ambiguous. This case is witnessed by grammar *G*2, where along with the introduction of base pair specific rules, another source of ambiguity is introduced.

Often, non-ambiguous grammars require more space in their implementation via a CYK parser. For example, the non-ambiguous Wuchty algorithm (RNAsubopt, [2]) requires four tables for storing intermediate results, while the ambiguous Zuker-Stiegler recurrences (Mfold, [7]) require only two. Two other cases in point are (a) and (b) below, while (c) shows that the non-ambiguous grammar can also be smaller.

Ambiguity can have many sources. Here, we present three common situations that lead us to write ambiguous rules, but can be easily avoided.

(a) Lists of adjacent elements of the same type, {*S*^*n*^}:

Consider *S *→ *SS*|*U *versus *L *→ *LS*|*S*, *S *→ *U*. The left-hand rule generates the language {*S*^*n*^} in an ambiguous way. For example, *S*^3 ^has the two derivations **S **→ *S***S **→ *SSS *and **S **→ **S***S *→ *SSS*, where the generating nonterminal symbol is written in bold face. By contrast, with the right-hand rules there is only the derivation **L **→ **L***S *→ **L***SS *→ *SSS*. The price for non-ambiguity is the new nonterminal symbol *L*, more parameters in the training set, and possibly another DP table in the implementation.

(b) Embedded elements, {*a*^*m*^*Ta*^*n*^}:

Consider *R *→ *aR*|*Ra*|*T *versus *R *→ *aR*|*V*, *V *→ *Va*|*T*.

For a given string *a*^*m*^*Ta*^*n*^, the first two alternatives of the left-hand rule produce the initial string *a*^*m *^and the terminal *a*^*n *^in arbitrary order, while the right-hand rules produce *a*^*m*^completely before *a*^*n*^, allowing for only one derivation. An analog case is the embedding {*a*^*m*^*Tb*^*n*^}. As above, an extra nonterminal symbol is required to achieve non-ambiguity.

(c) *ε*-rules, *L *→ *ε*:

Sometimes it is tempting to add a special case by using *ε*. Consider *L *→ *LS*|*S*|*ε*, which generates {*S*^*n *^|*n *≥ 0} by adding an *ε*-rule to the non-ambiguous rules in (a). Now, each string of length > 0 has two derivations, e.g. **L **→ **L***S *→ *S *and **L **→ *S*. The solution here is to drop the middle alternative, *L *→ *S*.

The general case of *ε*-rules may be more tricky to handle. In general, all context free languages can be described without *ε*-rules, except possibly one for the axiom symbol. However, if *ε*-rules were used relentlessly, eliminating them without affecting the language may require a major redesign of the grammar.

##### Degree of ambiguity and consequences for testing

Dowell and Eddy showed that semantic ambiguity produces sometimes mildly, sometimes drastically false results. In one experiment, they showed that the CYK algorithm for the semantically ambiguous grammar *G*1 does not give the optimal secondary structure for about 20% of a sample set of 2455 sequences. The same experiment for grammar *G*2 even gave a rate of 98% false results. The explanation of the difference in effect lies with the degree of ambiguity. The degree of ambiguity of a given annotation sequence is the number of its derivations, i.e. a degree of 1 means that this annotation sequence is not ambiguous. Depending on the involved productions, a particular string can have a constant, polynomial, or exponential number of derivations. The latter is the rule rather than the exception. It is easy to calculate for the left production rule of case (b) above that the sequence {*a*^*m*^*Ta*^*n*^} has  derivations starting from *S*. Moreover, if derivations emerging from *T *are also ambiguous, the degrees of ambiguity multiply.

Studying sources of ambiguity helps to better understand the nature of the error. Depending on the grammar, certain types of RNA structures may have their probability split up over a large number of derivations, while others are unaffected. This makes it difficult to judge the amount of testing required, and the confidence achieved with the approaches presented in the next section.

#### Testing for ambiguity

Performing a test for semantic ambiguity allows us to obtain more confidence in the grammar, although testing cannot prove non-ambiguity, but only ambiguity.

##### Algorithmic arsenal for ambiguity testing

First, we create several variants of the Inside and Viterbi algorithms, which are our algorithmic arsenal for testing. *G*l serves as the expository example here; for any other grammar, recurrences can be given in an analogous way:

Input: Sequence *x *= *x*_1 _... *x*_*n*_

Initialization: for 1 ≤ *i *≤ *n*

*S *(*i*, *i*) = *P*_*S*→*ε*_

Iteration: for 1 ≤ *i *≤ *j *≤ *n*



Scoring schemes:









 = 1

*P*_*V*→*α *_= 0 for all other rules

By different interpretations of the operations *H*, o and *P*, different scoring schemes can be plugged in. The recurrences may also be "conditioned" by annotating the symbol sequence *x *with a given annotation sequence *s *[1]. In that case, the rule *S *→ *aS* is only allowed when the bases involved are anno-tated to form a base pair in *s. *This version of the recurrences will be denoted by *G*_*s*_.

Using the first two scoring schemes, we obtain the Viterbi and the Inside algorithm. Using the other two, we obtain an algorithm for counting the number of derivations for the input string, and an algorithm for base pair maximization. Base pair maximization will not be used in the sequel, it is included only to indicate the swiftness of transition from SCFG modeling to other DP-based analyses. These algorithms are available at the accompanying website [8], where readers are welcome to practice their insight on ambiguity matters.

In the following, we write *G *(*σ*, *x*) for running the CYK parser based on grammar *G *with scoring scheme *σ *on input *x*.

We recalled above that the formal treatment of semantic ambiguity requires a canonical representation of the objects under study. For RNA secondary structures, there is an obvious choice, our annotation sequences in the widely used dot-bracket notation (cf. Figure 1). Each secondary structure (excluding pseudoknots) is uniquely represented by such a string. The scoring scheme *Dotbracket *makes the CYK algorithm report all the structures it has analyzed for a given input sequence by producing their annotation sequences.



Here, the objective function *H *merely collects lists of dot-bracket strings, each *P*_*V*→*α *_is a function adding dots or brackets to strings.  is string concatenation. *P*_*S*→*SS *_is also string concatenation, but has the unusual type *String *→ (*String *→ *String*), in order to fit into our recurrences smoothly. Here, o expects a dot-bracket string as its left argument, a function as its right argument, and applies the latter to the former. For example, the function calls *P*_*S*→*SS *_(*P*_*S*→*gSu *_(*P*_*S*→*ε*_)) (*P*_*S*→*aS *_(*P*_*S*→*ε*_)) generate the annotation sequence ". ()" for the symbol sequence "agu". The reader may verify (using the aforementioned website) that *G*1 (*Dotbracket*, "agu") = ["(.)","(.)",".()","...","...", etc.], where the duplicate entries result from the ambiguity of *G*1.For example, the annotation sequence "..." is found 48 times.

Using these algorithms in concert for some RNA sequence *x*, we obtain from *G*(*Viterbi, x*) the probability of the most likely derivation for *x*, from *G*(*Counting, x*) the number of possible derivations, and from *G*(*Dotbracket, x*) the complete list of the annotation sequences associated with these derivations – possibly containing duplicates in the case of semantic ambiguity.

##### Testing procedures

###### Brute force testing

Checking for duplicates in *G*(*Dotbracket, x*). We can simply enumerate the dot-bracket representation of all structures exhaustively for a given input string and check for any repeats. There are some duplicates in *G*(*Dotbracket, x*) if and only if *x *can fold into an ambiguous annotation sequence (which may be precluded by its nucleotide content). Performing this test on a large number of inputs *x *should give a good hint whether ambiguity is present. Of course, enumerating the annotation sequences for all possible derivation trees creates voluminous output, and the automated check for duplicates requires some careful programming. Hence, this test is practical only for short sequences.

###### Sampling structures from sample sequences

Test *G*(*Viterbi, x*) = *G*_*s*_(*Inside, x*)? Dowell and Eddy suggested a testing procedure that relies on a comparison of the results from the Viterbi and the Inside algorithms, where the latter is conditioned on the most likely annotation sequence *s *returned by the Viterbi run. *G*_*s*_(*Inside, x*) sums up probabilities over *all *derivations representing annotation sequence *s*. The tested equation therefore holds if and only if the annotation sequence *s *has exactly one derivation tree. If there are more than one, the Inside algorithm will return a higher probability than the Viterbi run, which indicates ambiguity of *s *(and hence *G*)*. *Similarly, *G*_*s*_(*Counting, x*) directly computes the number of derivations for *s*, where a result larger than 1 signals ambiguity.

Dowell and Eddy suggest to run the test also for a sample of suboptimal annotation sequences for *x*. As a variant, we can do the same test based on a *minimizing *Viterbi run (setting *H *= min). Since the minimizing Viterbi run gives us the least probable derivation tree, we may have a higher chance to find an ambiguous one (if present) than in the maximizing run.

In any case, this test works with samples of sub-optimal annotation sequences for a test set of sequences, and it is difficult to give general guidelines how much testing is required. The four grammars *G*3 - *G*6 passed the Dowell-Eddy test in [1], and in the next section we shall prove their non-ambiguity. In this sense, we can state that this test has already worked quite well in practice. However, the eternal dilemma of testing persists – only if we confirmed the above equation for *all x*, semantic non-ambiguity would be assured.

###### Structure counting for sample sequences

Test *G*(*Counting, x*) = *R*(*Counting, x*)? An even stronger test is possible when we have a reference grammar *R *available that generates the same language and is known to be semantically non-ambiguous. Grammar *G *will produce counts that are larger than those of *R *if and only if *G *allows ambiguous derivations for *x*. Still, if this test succeeds, this does not imply non-ambiguity of *G*. But this test is much more thorough than our previous one, as the entire structure space of each tested *x *is analyzed. For example, a sequence of length 30 has an expected number of 175550 feasible structures [3]. Thus, one run of this test has the testing power of 175550 runs of the previous one. Several non-ambiguous reference grammars for RNA are known – the critical part here is to assure that our grammar *G *to be tested describes the same language as *R*. Both grammars must impose the same restrictions on loop sizes, lonely base pairs, etc. This may be obvious in many cases, but in general, language equivalence is an undecidable problem in formal language theory.

###### Just-in-time testing

Test *G*(*Counting, x*) = *R*(*Counting, x*)? While testing cannot guarantee the non-ambiguity of the grammar, we can convert the previous idea to a test that ensures for each application run that the results are not affected by ambiguity. Prior to running *G*(*Viterbi, x*) for a given *x*, we test whether the property *G *(*Couniing, x*) = *R*(*Counting, x*)holds. This costs a constant factor in runtime, but solves the problem in the sense that when we get a positive test, we know the Viterbi result is correct for this input. If the grammar is ambiguous, this will be detected with the first application where it occurs.

#### Proving non-ambiguity

Proving the absence of ambiguity in a grammar is of course better than any test procedure.

#### Semantic ambiguity in dynamic programming is unde-cidable

Ambiguity of context free grammars is well-known to be algorithmically undecidable [9]. There exists no program that can determine for an arbitrary grammar *G *whether or not *G *is fl-ambiguous. Here, the problem is to decide whether a given SCFG is *se-mantically *ambiguous. It is not surprising that this problem is not easier:

#### Theorem 1 *Semantic ambiguity in dynamic programming is formally undecidable*

##### Proof

We show that for a given CFG *G *there exists a DP problem and an associated canonical model such that the DP algorithm is semantically am biguous if and only if the grammar is fl-ambiguous. Given an algorithm to decide ambiguity for DP problems, we could hence decide ambiguity for context free grammars, which is impossible. Details are given in the appendix. □

While this result rules out an automated proof procedure for arbitrary grammars used in SCFG modeling, there might still be the possibility to design such a procedure for a restricted class of grammars, say all grammars which describe RNA secondary structures. However, no such method is currently known.

#### Hand-made proof of non-ambiguity

A hand-made de-novo proof of the non-ambiguity of a new grammar *G *requires an inductive argument on the number of parses corresponding to the same annotation sequence. We constructed one such proof for the grammar published in [3]. It is not mathematically deep, but rather a tedious exercise, and the likelihood to produce errors or oversights is high. An easier approach is the use of a known, non-ambiguous "reference" grammar *R*, such that *L*(*G*) = *L*(*R*). By showing that a one-to-one mapping between parse trees of *G *and *R *exists, it is possible to prove the non-ambiguity of *G*. Such a proof remains manageable if the grammars are rather similar and the correspondence between derivations is easy to maintain. For grammars that are rather distinct, the proof is as messy as the de-novo proof.

#### Mechanical proof of non-ambiguity

We now present a mechanical technique that is a *partial *proof procedure for the case of modeling RNA structure with SCFGs: If it succeeds, it proofs non-ambiguity, if it fails, we do not know. We shall show that the method succeeds on several relevant grammars.

The technique described in the following comprises two steps. First, we remove the syntactic ambiguity of the grammar and reduce a possibly existent semantic ambiguity to fl-ambiguity. Then we use a parser generator to check the transformed grammar for fl-ambiguity. This test can prove non-ambiguity of a large number of grammars.

##### Ambiguity reduction

Paired bases can always also be unpaired – this creates the syntactic (good) ambiguity. For example, grammar *G*l has four rules of the form *S *→ *aS*, one for each base *A, C, G, U*, and six rules of the form *S *→ *aS* for the six valid base pairs. Used in concert, they create the "good" ambiguity that allows us to parse "CAAAG" either as "(...)" or as ".....".

Remember that the dot-bracket notation is a canonical representation for RNA secondary structure. For any *G*, we denote by G* the transformed grammar that arises when we replace base pairs a,  by "(" and ")", and other base symbols by ".". Take for example

*G*5 : *S *→ *aS *| *aS**S *| *ε *(11 productions), which is transformed to

*G*5* : *S *→ '.'*S *| '(*'S'*)' *S *| *ε *(3 productions).

This transformation removes the syntactic ambiguity of *G*5 by differentiating between paired and unpaired bases and reduces the semantic ambiguity -if present – to fl-ambiguity of *G*5*. Note that the transformation from *G *to G* works for any grammar for RNA structure, as long as we can identify the corresponding bases of a base pair.

### Theorem 2 Let G* be derived from G according to the above rules. Then, G* is fl-ambiguous if and only if G is semantically ambiguous

#### Proof

Every dot-bracket string describes exactly one possible secondary structure. If G* is fl-ambiguous, there exist different derivations in G* for the same dot-bracket string *z*. Then, for an RNA sequence *x *compatible with *z*, using the corresponding productions there are different derivations in *G *which represent the same secondary structure *z*. This is equivalent to semantic ambiguity of *G*. If G* is non-ambiguous, only a single derivation exists for every *z *in *L*(G*). A single derivation exists in *G *for a compatible RNA sequence *x*, and hence, *G *is semantically non-ambiguous. □

##### Non-ambiguity proof

By the transformation described above, the task of proving semantic non-ambiguity of *G *is transformed to the task of proving fl-non-ambiguity of G*. As stated above, this question is undecidable in general. However, compiler technology provides a partial proof procedure: If a deterministic parser can be generated for a grammar, then it is non-ambiguous [5]. We shall apply a parser generator to G*.

Simply speaking, a parser generator takes a file with a context free grammar as input, and generates a program which implements the parser for this grammar. This parser must be deterministic, and, in contrast to our CYK parsers, it only exists for non-ambiguous grammars. There are many such generators available; we will focus on the class of *LR*(*k*) grammars [10] and their parser generators. A context free grammar is called *LR*(*k*) if a deterministic shift reduce parser exists that uses *k *symbols of lookahead. By definition, an *LR*(*k*) grammar is non-ambiguous, and for a given *k *it is decidable whether a grammar is *LR*(*k*)*. *This decision can be assigned to a parser generator. Given the grammar and the lookahead *k*, a parser generator tries to construct a parser that uses *k *symbols of lookahead. When successful, the non-ambiguity of the grammar is proved. When the grammar is not *LR*(*k*), the generator will not be able to create a deterministic parser and reports this situations in form of "shift-reduce" and "reduce-reduce"-conflicts to the user. In this case, we do not know whether the parser generator might be successful for a larger *k*, and the question of ambiguity remains undecided.

##### Applications

For our experiments, we used the MSTA parser generator of the COCOM compiler construction toolkit [11]. MSTA is capable of generating *LR*(*k*) parsers for arbitrary *k. *Note that compiler writers prefer other parser generators like yacc [12] and bison [13], which for efficiency reasons only implement *LR*(1) parsers. We, however, are not planning to run the parser at all. Its successful construction is the proof of non-ambiguity; for applying our SCFG, we need the original grammar and its CYK parser.

MSTA accepts input files in the widely used yacc format. The following shows the input file for grammar *G*5:



Feeding this file into MSTA with *k *= 1 yields a deterministic shift-reduce parser for grammar *G*5. This proves that *G5 *is *LR*(1), has a deterministic *LR*(1) parser, and is therefore non-ambiguous.

Table 1 summarizes the results for grammars *G*1 to *G*6. For *G*1 and *G*2, the results only show that both grammars are not *LR*(1), *LR*(2) or *LR*(3). Although no real proof, the magnitude and growth of the number of conflicts with increasing *k *gives a strong hint at the ambiguity of these grammars.

**Table 1:** Results of mechanical proof procedure. Number of shift-reduce (SR) and reduce-reduce (RR) conflicts when feeding example grammars G1 to G8 into parser generator MSTA. A 0/0 entry indicates a successful proof of non-ambiguity. Note that for increasing *k*, the number of conflicts may remain constant or even grow before it goes down to 0/0.

Grammar	k	SR/RR conflicts
G1	1	24/12
G1	2	70/36
G1	3	195/99
G2	1	25/13
G2	2	59/37
G2	3	165/98
G3	1–3	4/0
G3	4	16/0
G3	5	0/0
G4	1	0/0
G5	1	0/0
G6	1	0/0
G7	1–6	5/0
G7	7	0/0
G8	1	0/0

Grammar *G*3 is *LR*(5) and *G*4 to *G*6 are *LR*(1). Therefore, we have proved mechanically that the four "good" grammars studied by Dowell and Eddy are definitely non-ambiguous. The two additional grammars *G*7 and *G*8 from [1], not reproduced here, were also included in the study and proved to be non- ambiguous.

In Table 1 we also report on the number of conflicts found by the parser generator for increasing values of *k*. While the nature of these conflicts is not relevant for us, the table shows that various behaviors are possible. Their numbers may grow (*G*3) or may remain constant (*G*7) before they go to zero for some *k*.

##### Experience from a larger example

The parser generator test works quite well for the small grammars we presented so far. However, there exist cases where, due to the finite lookahead of the generated parser, the parser generator reports conflicts while the grammar is in fact non-ambiguous. In the following, we report on one such case, and show how to deal with this situation.

**Figure 2:**
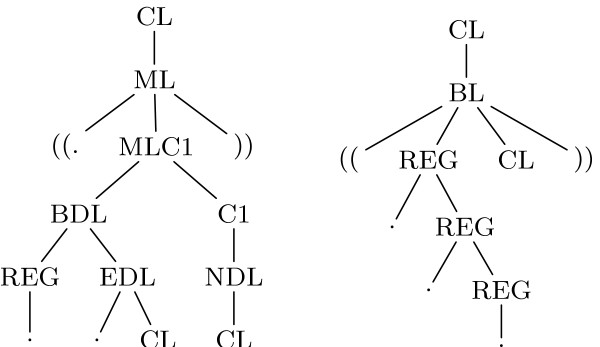
**Two example derivations**. Two example derivations of a grammar taken from [14]. The left side is part of a multiloop derivation, the right side part of a left bulge.

In his thesis [14], Björn Voss introduced a new grammar that promises to handle dangling bases of multiloop components in a non-ambiguous way. With 28 nonterminal symbols and 79 rules, the grammar is quite large. In such a case, mechanical assistance is strongly required. Our first approach with the parser generator succeeded, except for one small part of the grammar for which it reports a con-flict. Figure 2 shows two example derivations where this conflict occurs.

The central nonterminal of the grammar is CL, which splits up into closed structures like hairpin loops, bulges, and multiloops. Due to the necessity to handle dangling bases in a non-ambiguous way, the rules for multiloops are the most complicated of this grammar. Altogether, 11 nonterminals and 35 rules are used exclusively for this purpose. The construction of these rules guarantees, that every derivation of a multiloop *must *lead to at least two closed substructures. One of these derivations is shown on the left side of Figure 2. Therefore, a derivation of a multiloop can by no means conflict with a derivation of a left bulge, which must include a *single *closed substructure. However, the parser generator runs into a conflict here. Consider the following annotation sequence:



Here, the string "((...((" appears two times in the annotation sequence. The first appearance denotes a left bulge, the second the beginning of a multiloop. The decision which of these two is given can only be made after the first closed substructure is completely processed. Since the generated parser can only read a limited number of input characters ahead (k), the parser generator is not able to construct a deterministic parser for this situation and reports a conflict.

However, we can circumvent this problem by extending the alphabet of the annotation sequence by an additional character (say, ':') for unpaired bases in left bulges^1^:



Since a multiloop's derivation can not conflict with that of a bulge, this modification does not alter the ambiguity or non-ambiguity of the grammar. The important difference is that positional information is turned into symbolic information.

After this modification, the parser generator runs smoothly through the grammar, which proves its non-ambiguity.

## Conclusion

In this work, we have presented testing methods and a partial proof procedure to analyze the semantic ambiguity of SCFGs. We have shown that the problem is not decidable for dynamic programming over sequence data in general, and that hence there is no standard solution that works for all cases. It remains open whether specifically for the class of grammars that describe RNA secondary structure, this problem is decidable. We have proposed several tests, and a partial, mechanical proof procedure. We mechanically proved that the six grammars that passed Dowell and Eddy's test for non-ambiguity are actually non-ambiguous. We also reported on a proof of the non-ambiguity of a new and large grammar for RNA secondary structures, whose sophistication makes it inadvisable to rely solely on human reasoning.

We want to point out that the non-ambiguity proofs for the grammars studied here do not solve the problem of ambiguity for modeling of RNA secondary structures once and for all. New scientific interests and research questions will always demand new grammars. An example is a grammar that is restricted to a special class of structures of an RNA family. This allows us to define a thermodynamic matcher, which uses the minimum free energy as a scoring scheme and focuses only on a specific realm of secondary structures. Here, for every new RNA family, a new grammar must be devised. This demonstrates a continuous need for new, specialized grammars. Every time we develop a new grammar, the dragon of ambiguity raises its head, but with the weapons presented here, we can be confident to defeat it.

## Methods

Our way to describe various tests by combining a grammar with varying scoring schemes is derived from the algebraic dynamic programming method, described in detail in [6]. The theoretical framework of this method also underlies the proof of Theorem 1. The parser generator MSTA used as a partial proof method is available at [11].

## Authors' contributions

RG suggested the topic and contributed the undecid-ability proof. JR worked out the testing procedures and PS the mechanical proof procedure. All authors cooperated closely in writing the manuscript.

## Appendix: Ambiguity in DP is undecidable

Dynamic programming is a very general programming technique, and its scope is not precisely circumscribed. We prove our undecidability result for the well defined class of algebraic dynamic programming [6] problems, which of course implies undecidability in general. Simply speaking, a DP problem is given by a grammar *G *and a scoring scheme *σ *(not necessarily stochastic), as was exemplified in Section *Testing for ambiguity*.

### Theorem 1 *Semantic ambiguity in dynamic programming is formally undecidable*

#### Proof

For an arbitrary context free grammar *G*, we can construct a DP problem where *L*(*G*) serves as the canonical model, and show that the context free grammar *G *is ambiguous if and only if the DP problem is semantically ambiguous.

Let *G *be a context free grammar. Without loss of generality, we can assume that each production is either of the form *A *→ *t*, generating a terminal symbol, or of *A*_0 _→ *A*_1_...*A*_*n*_, *n *≥ 0, generating a series of nonterminal symbols. We construct a scoring scheme *σ *for grammar *G *such that *G*(*σ*, *x*) computes all derivation trees for *x*. Similar to the scoring scheme *Dotbracket*, we set *H *= collect and *x*o*f *= *f*(*x*). For each production *π *we use a unique tree label *T*_*π*_. We define ...*A*_*n *_(*a*_*n*_)...(*a*_1_) = ...*A*_*n *_(*a*_1_,...,*a*_*n*_), and *P*_*A*→*t *_= *t*.



By construction, *G*(*σ*, *x*) constructs the list of all derivation trees for *x*. The canonical mapping *ν *(from derivation trees to their derived strings) is simply given by *ν*(...*A*_*n *_(*a*_1_,...,*a*_*n*_)) = *ν*(*a*_1_)...*ν*(*a*_*n*_) and *ν*(*t*) = *t*. By construction, the domain of *v *are the derivation trees of *G*, its range is *L*(*G*)*. *Hence, *v *is injective if and only if *G *is non-ambiguous. Could we formally decide the semantic ambiguity of an arbitrary DP problem, we could do so for the problem given by *G *and *σ*, and hence, ambiguity of context free languages would be decidable. □

## Note

^1^For the same reason, this modification is also necessary in the rules for internal loops.
